# A high-affinity, partial antagonist effect of 3,4-diaminopyridine mediates action potential broadening and enhancement of transmitter release at NMJs

**DOI:** 10.1016/j.jbc.2021.100302

**Published:** 2021-01-17

**Authors:** Kristine S. Ojala, Scott P. Ginebaugh, Man Wu, Evan W. Miller, Gloria Ortiz, Manuel Covarrubias, Stephen D. Meriney

**Affiliations:** 1 Department of Neuroscience, Center for Neuroscience, University of Pittsburgh, Pittsburgh, Pennsylvania, USA; 2 Departments of Chemistry and Molecular & Cell Biology, University of California, Berkeley, Berkeley, California, USA; 3 Department of Neuroscience, Thomas Jefferson University, Philadelphia, Pennsylvania, USA

**Keywords:** neurotransmitter release, neuromuscular junction (NMJ), voltage-gated potassium (Kv) channel, voltage-gated calcium (Cav) channel, action potential (AP), neurological disease, Lambert–Eaton myasthenic syndrome (LEMS), 3,4-DAP, 3,4-diaminopyridine, AP, action potential, BeRST, Berkeley red-based sensor of transmembrane potential, BTX, alpha-bungarotoxin, Cav, voltage-gated calcium, EPP, endplate potential, FWHM, full width at half maximum, Kv, voltage-gated potassium, LEMS, Lambert–Eaton myasthenic syndrome, mEPP, miniature endplate potential, NMJ, neuromuscular junction, QC, quantal content, ROI, region of interest

## Abstract

3,4-Diaminopyridine (3,4-DAP) increases transmitter release from neuromuscular junctions (NMJs), and low doses of 3,4-DAP (estimated to reach ∼1 μM in serum) are the Food and Drug Administration (FDA)-approved treatment for neuromuscular weakness caused by Lambert–Eaton myasthenic syndrome. Canonically, 3,4-DAP is thought to block voltage-gated potassium (Kv) channels, resulting in prolongation of the presynaptic action potential (AP). However, recent reports have shown that low millimolar concentrations of 3,4-DAP have an off-target agonist effect on the Cav1 subtype (“L-type”) of voltage-gated calcium (Cav) channels and have speculated that this agonist effect might contribute to 3,4-DAP effects on transmitter release at the NMJ. To address 3,4-DAP’s mechanism(s) of action, we first used the patch-clamp electrophysiology to characterize the concentration-dependent block of 3,4-DAP on the predominant presynaptic Kv channel subtypes found at the mammalian NMJ (Kv3.3 and Kv3.4). We identified a previously unreported high-affinity (1–10 μM) partial antagonist effect of 3,4-DAP in addition to the well-known low-affinity (0.1–1 mM) antagonist activity. We also showed that 1.5-μM DAP had no effects on Cav1.2 or Cav2.1 current. Next, we used voltage imaging to show that 1.5- or 100-μM 3,4-DAP broadened the AP waveform in a dose-dependent manner, independent of Cav1 calcium channels. Finally, we demonstrated that 1.5- or 100-μM 3,4-DAP augmented transmitter release in a dose-dependent manner and this effect was also independent of Cav1 channels. From these results, we conclude that low micromolar concentrations of 3,4-DAP act solely on Kv channels to mediate AP broadening and enhance transmitter release at the NMJ.

Lambert–Eaton myasthenic syndrome (LEMS) is a neuromuscular disease caused by an autoantibody-mediated attack on the presynaptic Cav2.1 type (also called “P/Q type”) voltage-gated calcium (Cav) channels and other presynaptic proteins at neuromuscular junctions (NMJs) (1, 2, 3, 4, 5, 6). The resulting antibody-mediated loss of proteins associated with transmitter release sites results in a reduction of acetylcholine release from the NMJ and leads to a failure of some postsynaptic muscle fibers to initiate an action potential (AP), leading to a weaker muscle contraction. Clinical and animal model studies suggest that neuromuscular transmission, and subsequently muscle strength, can be improved by the use of 3,4-diaminopyridine (3,4-DAP), which is a small molecule that acts as an antagonist at voltage-gated potassium (Kv) channels. 3,4-DAP was recently approved by the FDA to treat LEMS (7, 8, 9, 10) and has been shown to be effective at increasing neuromuscular strength in patients with LEMS (11, 12, 13, 14). However, 3,4-DAP has dose-dependent side effects that restrict the amount that patients take to relatively small doses, which prevents full symptomatic relief in many patients with LEMS (15, 16). Patients are typically prescribed 10- to 20-mg oral doses of 3,4-DAP to be taken several times during the day and report peak clinical effects for 3 to 8 h after each dose (17). 3,4-DAP has been reported to have a serum half-life of 1 to 3 h, and pharmacokinetic studies cite peak serum concentrations of ∼40 to 110 ng/ml after a 20-mg oral dose (18, 19, 20, 21, 22). Similar doses of 3,4-DAP have also been used off-label for a variety of other neuromuscular weakness conditions, including congenital myasthenic syndrome (23, 24, 25, 26, 27, 28), muscle-specific receptor tyrosine kinase myasthenia gravis (29), downbeat nystagmus (30), and multiple sclerosis (31, 32, 33, 34).

The mechanism of action of 3,4-DAP at neuromuscular synapses is canonically thought to be a block of Kv3 (also called “A-type”) channels. Kv3.3 and Kv3.4 channels are the subtypes selectively localized at mammalian neuromuscular motor nerve terminals (35) and are thought to be predominantly responsible for speeding the repolarization of the presynaptic AP. By blocking Kv3 channels, 3,4-DAP is hypothesized to broaden the presynaptic AP duration, thus indirectly increasing calcium ion flux by increasing the number of presynaptic Cav (Cav2) channels that open during an AP. Because calcium-triggered acetylcholine release is nonlinearly dependent on the calcium concentration in nerve terminals, a relatively small increase in calcium ion entry can generate a much larger increase in neurotransmitter release (36).

Recent investigations have challenged the conventional mechanism of action of aminopyridines. First, prior concentration-response studies of aminopyridine action on Kv channels have often been restricted to the use of 4-aminopyridine and yielded an IC_50_ between 30 μM and 2.5 mM depending on the types of potassium channels expressed (37, 38, 39, 40, 41, 42, 43, 44, 45, 46), with a high sensitivity to 4-aminopyridine reported for Kv3 channels (80-μM IC_50_; (47)). Because 4-aminopyridine crosses the blood–brain barrier better than 3,4-DAP, the latter has been preferred for the treatment of peripheral neuromuscular diseases (48, 49). Therapeutic concentrations of 3,4-DAP are predicted to be in the low micromolar range, and 3,4-DAP has been reported to have significant effects on squid giant axon potassium channels at these concentrations (50). Second, a direct agonistic action of 3,4-DAP on Cav1 type (also called “L-type”) channels was reported (41, 51). However, the clinical relevance of the reported effects of 3,4-DAP on Cav channels was debated because the 3,4-DAP concentrations evaluated in these studies were significantly above blood serum levels found in LEMS treatment conditions (52, 53). Furthermore, because Cav1 channels usually lack the synaptic protein interaction sites present in Cav2 channels (54, 55, 56), Cav1 channels are thought to reside outside of synaptic vesicle release sites in the NMJ and therefore are not thought to directly control acetylcholine release (as Cav2 channels do) at healthy synapses. However, it is possible that Cav1 channels may have a minor role at neuromuscular synapses that is revealed under pharmacological conditions (57, 58, 59, 60, 61), and Cav1 channels may have a compensatory contribution to the control of transmitter release in diseased conditions such as LEMS (61, 62).

Therefore, to investigate the physiological mechanism accounting for the clinical response to 3,4-DAP, we tested the effects of a therapeutic concentration of 3,4-DAP (1.5 μM) on (a) peak currents of Kv3 channels expressed in HEK293T cells, (b) the presynaptic AP waveform at frog and mouse motor nerve terminals, and (c) transmitter release from weakened frog and mouse NMJs. To explore the role of Cav1 channels in 3,4-DAP–mediated effects at NMJs, we conducted our transmitter release and AP experiments in the presence or absence of a Cav1 antagonist (nitrendipine) and compared the results. In addition, we examined the effects of a supratherapeutic concentration of 3,4-DAP (100 μM) to allow direct comparisons with prior studies that used this higher concentration (41, 51, 53). For the purpose of this report, we define a supratherapeutic concentration as one that is about 100-fold higher (100 μM) than the measured concentration in the serum of patients with LEMS after taking the typical prescribed dose of 3,4-DAP (18, 19, 20, 21, 22).

Our results demonstrate that the therapeutic concentration of 1.5-μM 3,4-DAP has a small but significant effect on both Kv3.3 and Kv3.4 channels, and we show that this concentration broadens the presynaptic AP waveform to increase the magnitude of neuromuscular transmission independent of a Cav1 contribution. The effect of the supratherapeutic concentration of 100-μM 3,4-DAP was more pronounced, but broadening of the AP and the increase in the magnitude of transmitter released remained independent of effects on Cav1 channels. These results support the hypothesis that the clinical effects of 3,4-DAP in the treatment of LEMS are caused by a partial block of Kv channels, independent of any effects of Cav1 channels.

## Results

### 3,4-DAP effects on Kv3 potassium channels

**Figure 1:**
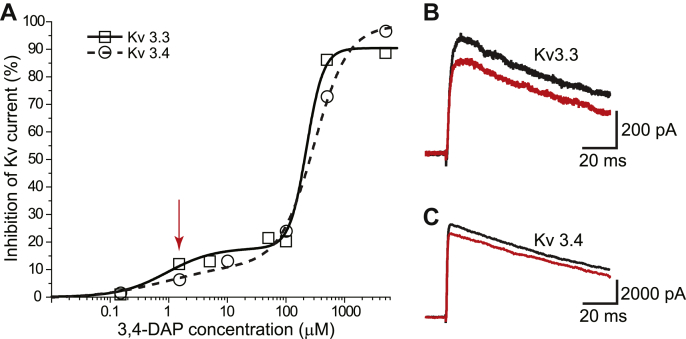
**Concentration-dependent effects of 3,4-DAP on Kv3.3 and Kv3.4 potassium currents expressed in HEK293T cells.***A*, plot of the inhibition of current through Kv3.3 (*open squares*, *solid fit line*) and Kv3.4 (*open circles*, *dashed fit line*) channels after exposure to varying concentrations of 3,4-DAP; *n* = 3 to 6. The *red arrow* indicates the data at 1.5-μM 3,4-DAP concentration for which sample currents are shown in panels *B* and *C*; SD bars are smaller than the symbol sizes. *B*, sample Kv3.3 currents activated by a voltage step from −100 mV to +40 mV and shown before (*black trace*) and after (*red trace*) exposure to 1.5-μM 3,4-DAP. *C*, sample Kv3.4 currents activated by a voltage step from −100 mV to +40 mV and shown before (*black trace*) and after (*red trace*) exposure to 1.5-μM 3,4-DAP. 3,4-DAP, 3,4-diaminopyridine; Kv, voltage-gated potassium.

When considering which subtypes of Kv channels might be blocked by 3,4-DAP within mammalian motor nerve terminals, we were guided by prior work at the mouse NMJ which demonstrated that Kv3.3 and Kv3.4 were the subtypes detected by immunohistochemistry (35). To determine the concentration-dependent effects of 3,4-DAP on these channel types, we expressed Kv3.3 and Kv3.4 channels in HEK293T cells and used whole-cell patch-clamp electrophysiology to measure the change in current after 3,4-DAP application. Using a 100- or 500-ms step depolarization protocol (−100 mV to +40 mV), we activated Kv3.3 or Kv3.4 current and then measured the peak current before and after application of 3,4-DAP (at concentrations ranging between 0.15 and 5000 μM). We found a concentration-dependent block of both Kv3.3 and Kv3.4 currents that was similar for each channel subtype. Importantly, the therapeutic concentration of 3,4-DAP (1.5 μM) significantly reduced Kv3.3 and Kv3.4 currents by about 10% (Fig. 1). We observed that the concentration-response relationship appeared to be best fit by a biphasic Hill equation (Fig. 1; Prism, GraphPad). For Kv3.3 and Kv3.4, the high affinity fit yielded IC_50_ values of 2.5 and 10.3 μM and Hill coefficients of 0.7 and 0.6, respectively. Because the maximum inhibition for this high-affinity activity was approximately 20 to 25%, 3,4-DAP binding to high affinity sites on Kv3.3 and Kv3.4 exhibited partial antagonist activity. The low-affinity fits of the Kv3.3 and Kv3.4 data yielded IC_50_ values of 151 and 231 μM and Hill coefficients of 3.0 and 1.4, respectively. The maximum inhibition for this low-affinity activity was near 100%.

Because we were interested in a therapeutic concentration of 3,4-DAP with respect to its effect on calcium-triggered transmitter release at the NMJ, we confirmed that 1.5-μM 3,4-DAP had no effect on Cav2.1 or Cav1.2 current. When comparing the peak and integral of calcium current before and after application of 1.5 μM 3,4-DAP, we found no significant effects on either Cav2.1 (drug/control = 1.04 ± 0.08 peak; 1.01 ± 0.09 integral; *n* = 3; mean ± SD) or Cav1.2 (drug/control = 0.95 ± 0.21 peak; 0.94 ± 0.08 integral; *n* = 3; mean ± SD) currents.

### 3,4-DAP effects on the presynaptic AP waveform at the NMJ

The mechanism of action underlying the effects of 3,4-DAP at the NMJ is canonically thought to be due to a partial block of presynaptic Kv channels leading to a broadening of the presynaptic AP. To date, no studies have directly measured 3,4-DAP–mediated effects on the presynaptic AP waveform at the NMJ. Thus, we used a voltage-sensitive fluorescent dye (Berkeley red-based sensor of transmembrane potential [BeRST] 1; (63)) to directly measure the impact of 3,4-DAP on the duration of the presynaptic AP waveform at frog and mouse NMJs. The BeRST 1 dye is fast enough to resolve the AP and has been shown to not affect the electrical properties or AP waveforms of neurons (63). We performed a paired experiment where the control AP waveform was recorded from a single nerve terminal, which was then was exposed to 3,4-DAP for 30 min before the AP waveform was recorded again at the same nerve terminal. To determine the duration of the presynaptic AP waveform, we measured the full width at half maximum (FWHM) of the recorded AP waveforms. This technique was performed in separate neuromuscular preparations in either vehicle or nitrendipine conditions to determine if blocking Cav1 channels affected the 3,4-DAP–mediated effects on the AP waveform.

To ensure that AP duration was not altered by prolonged experimental time in the imaging setup, we performed the imaging procedure on control experiments without 3,4-DAP over the same time course as a typical 3,4-DAP experiment. We found no significant changes in AP duration during these control experiments, demonstrating that our imaging procedure itself was not impacting the AP waveform (data not shown).

**Figure 2:**
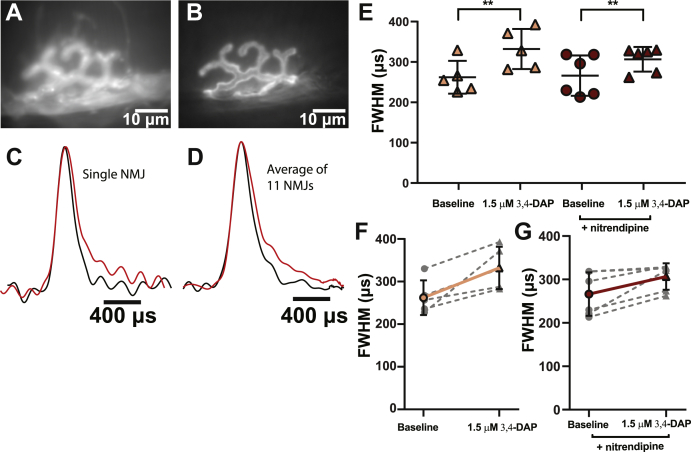
**Therapeutic concentrations of 3,4-DAP broaden the presynaptic AP waveform independent of Cav1 channels at the mammalian NMJ.***A*, a BeRST 1 dye–stained image of a mammalian presynaptic motor nerve terminal. *B*, an Alexa Fluor 488 BTX–stained image of the same terminal as in panel *A*. *C*, normalized splines of presynaptic AP waveforms recorded from a single nerve terminal before (*black*) and after (*red*) the addition of 1.5-μM 3,4-DAP. *D*, the normalized average of all pre-drug (*black*) and post 1.5-μM 3,4-DAP (*red*) presynaptic AP waveform splines recorded from mammalian motor nerve terminals (*n* = 11). *E*, FWHMs of recorded AP waveforms before (*circles*) or after (*triangles*) 1.5-μM 3,4-DAP application to vehicle (*peach*) or nitrendipine (*red*) treated mouse NMJs. 1.5-μM 3,4-DAP significantly broadens the AP waveform independent of nitrendipine (two-way mixed ANOVA: there was a significant main effect of 1.5-μM 3,4-DAP (F (1,9) = 22.40, ∗∗*p* = 0.0011), but no main effect of nitrendipine (F (1,9) = 0.2139, *p* = 0.6547), or interaction between 1.5-μM 3,4-DAP and nitrendipine (F (1,9) = 1.583, *p* = 0.2399); vehicle, *n* = 5; nitrendipine, *n* = 6). *F*-*G*, plots of individual paired recorded values (*gray dotted lines*) of AP duration (FWHM) before and after application of 1.5-μM 3,4-DAP with a superimposed average (vehicle, *solid peach line*; nitrendipine, *solid red line*). 3,4-DAP, 3,4-diaminopyridine; AP, action potential; BeRST, Berkeley red-based sensor of transmembrane potential; BTX, alpha-bungarotoxin; Cav, voltage-gated calcium; FWHM, full width at half maximum; NMJ, neuromuscular junction.

We first measured the impact of a therapeutic concentration of 1.5-μM 3,4-DAP on the duration of the presynaptic AP waveform at mouse motor nerve terminals (Fig. 2). We found that 1.5-μM 3,4-DAP broadened the presynaptic AP in vehicle-treated mouse NMJs, increasing the FWHM of the AP waveform from 262.2 ± 40.7 μs to 332.2 ± 49.6 μs. The presence of nitrendipine did not significantly alter the impact of 1.5-μM 3,4-DAP, with the FWHM of the AP waveform in the nitrendipine-treated mouse NMJs increasing from 266.1 ± 50.0 μs to 306.7 ± 30.5 μs after the application of 1.5-μM 3,4-DAP (Fig. 2*E*).

**Figure 3:**
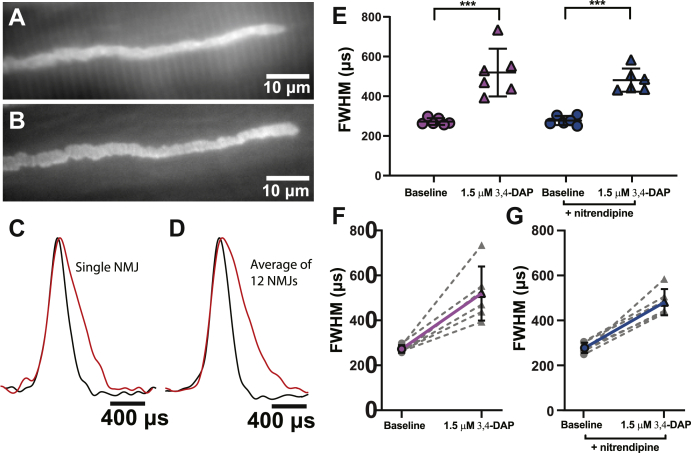
**Therapeutic concentrations of 3,4-DAP broaden the presynaptic AP waveform independent of Cav 1 channels at the frog NMJ.***A*, a BeRST 1 dye–stained image of a frog presynaptic motor nerve terminal. *B*, an Alexa Fluor 488 BTX–stained image of the same terminal as in *A*. *C*, normalized presynaptic AP waveform splines recorded from a single nerve terminal before (*black*) and after (*red*) the addition of 1.5-μM 3,4-DAP. *D*, the normalized average of all pre-drug (*black*) and post–1.5-μM 3,4-DAP (*red*) presynaptic AP waveform splines recorded from all frog motor nerve terminals (*n* = 12). *E*, FWHMs of recorded AP waveforms before (*circles*) or after (*triangles*) 1.5-μM 3,4-DAP application to vehicle (*pink*) or nitrendipine (*blue*) treated frog NMJs. 1.5 μM 3,4-DAP broadens the AP waveform independent of nitrendipine (two-way mixed ANOVA: there was a significant main effect of 1.5-μM 3,4-DAP (F (1,10) = 77.68, ∗∗∗*p* < 0.0001), but no main effect of nitrendipine (F (1,10) = 0.2967, *p* = 0.5979), and no significant interaction between 1.5-μM 3,4-DAP and nitrendipine (F (1,10) = 0.7278, *p* = 0.4136); vehicle, *n* = 6; nitrendipine, *n* = 6). *F*-*G*, plots of individual paired recorded values (*gray dotted lines*) of AP duration (FWHM) before and after application of 1.5-μM 3,4-DAP with a superimposed average (vehicle, *solid pink line*; nitrendipine, *solid blue line*). BTX, alpha-bungarotoxin; Cav, voltage-gated calcium; 3,4-DAP, 3,4-diaminopyridine; AP, action potential; BeRST, Berkeley red-based sensor of transmembrane potential; FWHM, full width at half maximum; NMJ, neuromuscular junction.

We next investigated the impact of 1.5-μM DAP on the AP waveform at frog motor nerve terminals to determine if the effects of 3,4-DAP on the AP waveform are conserved across species (Fig. 3). We found that 1.5-μM 3,4-DAP broadened the presynaptic AP in vehicle-treated frog NMJs, increasing the FWHM of the AP waveform from 272.5 ± 16.9 μs to 519.5 ± 120.5 μs. Again, we found no significant impact of the presence of nitrendipine on the effect of 1.5-μM 3,4-DAP, with the FWHM of the AP waveform in the nitrendipine-treated frog NMJs increasing from 277.9 ± 22.9 μs to 481.3 ± 58.3 μs after the application of 1.5-μM 3,4-DAP (Fig. 3*E*).

**Figure 4:**
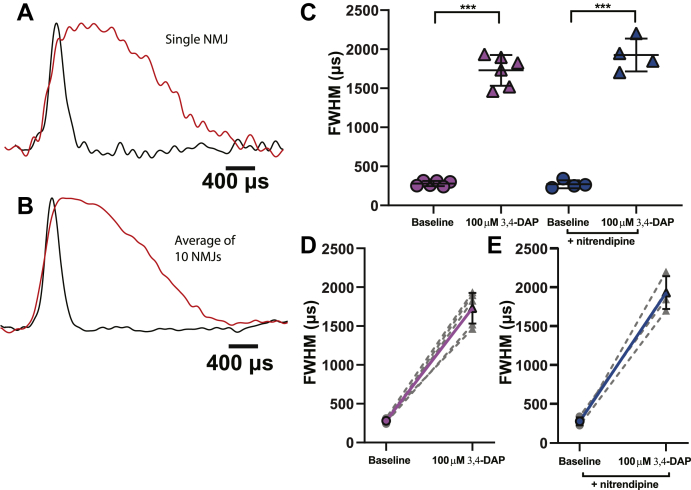
**A supratherapeutic concentration of 100-μM 3,4-DAP significantly broadens the frog presynaptic AP waveform independent of Cav1 channels.***A*, normalized presynaptic AP waveform splines recorded from a nerve terminal before (*black*) and after (*red*) the addition of 100-μM 3,4-DAP. *B*, the normalized average of all predrug (*black*) and post–100-μM 3,4-DAP (*red*) presynaptic AP waveform splines recorded from all frog motor nerve terminals (*n* = 10). *C*, FWHMs of recorded AP waveforms before (*circles*) or after (*triangles*) 100-μM 3,4-DAP application to vehicle (*pink*) or nitrendipine (*blue*) treated frog NMJs. 100-μM 3,4-DAP broadens the AP waveform independent of nitrendipine (two-way mixed ANOVA: significant main effect of 100-μM 3,4-DAP (F (1,8) = 524.7, ∗∗∗*p* < 0.0001), but no main effect of nitrendipine (F (1,8) = 2.035, *p* = 0.1916), and no interaction between 3,4-DAP and nitrendipine (F (1,8) = 2.292, *p* = 0.1685); vehicle, *n* = 6; nitrendipine, *n* = 4). *D* and *E*, plots of individual paired recorded values (*gray dotted lines*) of AP duration (FWHM) before and after application of 1.5-μM 3,4-DAP with a superimposed average (vehicle, *solid pink line*; nitrendipine, *solid blue line*). 3,4-DAP, 3,4-diaminopyridine; AP, action potential; Cav, voltage-gated calcium; FWHM, full width at half maximum.

Finally, we tested the impact of a supratherapeutic concentration (100 μM) of 3,4-DAP on the duration of the presynaptic AP waveform at the frog NMJ (Fig. 4). In vehicle-treated frog NMJs, 100-μM 3,4-DAP broadened the duration of the AP waveforms from an FWHM of 280.4 ± 32.6 μs to 1729.7 ± 197.0 μs. Even at this higher concentration of 3,4-DAP, we did not see any significant impact of nitrendipine on the 3,4-DAP–mediated broadening of the presynaptic AP waveform. The FWHM of the AP waveform in the nitrendipine-treated frog NMJs increased from 270.9 ± 51.1 μs to 1925.4 ± 210.3 μs after the application of 100-μM 3,4-DAP (Fig. 4*C*).

These data demonstrate that 3,4-DAP increases the duration of the presynaptic AP waveform at mammalian and frog NMJs in a dose-dependent manner, and that Cav1 calcium channels have no interaction with this effect. Because small changes in the duration of the AP waveform can greatly increase calcium flux and transmitter release at the NMJ (36, 64), these data further support the hypothesis that broadening of the presynaptic AP *via* the blocking of Kv3 channels is the primary mechanism by which 3,4-DAP increases transmitter release *in vivo*.

### 3,4-DAP increases neuromuscular transmission in a dose-dependent manner independent of Cav1 calcium channels

To characterize the dose-dependent effects of 3,4-DAP on weakened transmitter release at the NMJ, we used paired intracellular microelectrode recordings in *ex vivo* neuromuscular preparations to measure endplate potentials (EPPs) in response to nerve-evoked APs, both before and after exposure to either therapeutic (1.5 μM) or supratherapeutic (100 μM) concentrations of 3,4-DAP. In addition, we measured spontaneous miniature EPPs (mEPPs) from the same population of muscle fibers to determine quantal content (QC). We performed both EPP and mEPP recordings in the presence or absence of the Cav1 blocker nitrendipine to test the hypothesis that Cav1 calcium channels are important for 3,4-DAP effects.

We reduced the magnitude of transmitter release by performing all recordings in the presence of low concentrations of the calcium channel antagonist ω-agatoxin IVA (for Cav 2.1 channels at mouse NMJs) or ω-conotoxin GVIA (for Cav 2.2 channels at frog NMJs). Reducing transmitter release magnitude after exposure to submaximal concentrations of these toxins mimics the effect of neuromuscular diseases that weaken NMJs and importantly minimizes complications during data analysis because of nonlinear summation, ensuring that correction for nonlinear summation is accurate (65). In the absence of these selective Cav2 calcium channel blockers, control EPPs average 10 to 40 mV in amplitude above resting membrane potential (*e.g.*, from −70 mV resting membrane potential to a peak of −60 to −30 mV) at mouse and frog NMJs (6, 66). After exposure to 3,4-DAP (especially the 100-μM concentration), the EPP size can approach the acetylcholine receptor channel reversal potential (−10 mV), making 3,4-DAP–induced changes in transmitter release difficult to interpret and analyze. For these reasons, all experiments were performed using submaximal concentrations of a calcium channel blocker to reduce QC, allowing a more accurate assessment of 3,4-DAP effects on weakened neuromuscular transmission.

**Figure 5:**
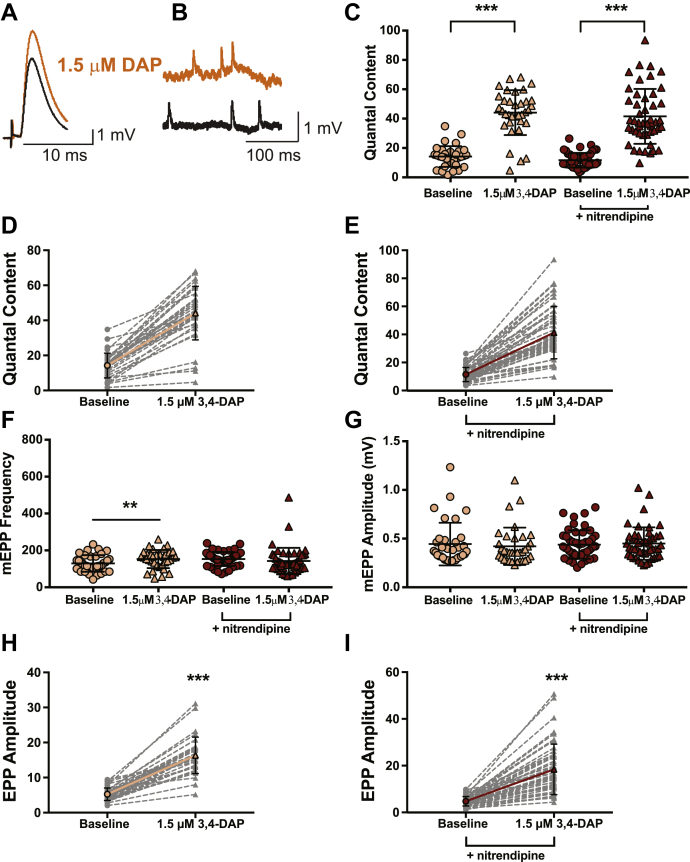
**1.5-μM 3,4-DAP dose dependently increases neuromuscular transmission independent of Cav1 channels in mouse neuromuscular junctions.***A* and *B*, sample traces of electrophysiological recordings of EPPs (*A*) and mEPPs (*B*) before and after 1.5-μM 3,4-DAP application. *C*, quantified quantal content before (*circles*) or after (*triangles*) 1.5-μM 3,4-DAP application to vehicle (*peach*) or nitrendipine (*red*) treated mouse NMJs. Two-way mixed ANOVA was used (there was a significant main effect of 1.5-μM 3,4-DAP (F (1, 81) = 347.5, ∗∗∗*p* < 0.0001); no significant main effect of nitrendipine (F (1,81) = 1.136, *p* = 0.2897) or interaction between 3,4-DAP and nitrendipine (F (1,81) = 0.0002, *p* = 0.9887); vehicle, *n* = 35; nitrendipine *n* = 48). *D* and *E*, plots of individual paired values (*gray dotted lines*) with a superimposed average (*solid peach line*, vehicle; *solid red line*, nitrendipine). *F*, there was a significant interaction between the effects of 1.5-μM 3,4-DAP and nitrendipine on mEPP frequency (two-way mixed ANOVA; no main effect of nitrendipine; F (1,81) = 0.4209, *p* = 0.5183) or 3,4-DAP; F (1,81) = 1.634, *p* = 0.2048), but a significant interaction between 3,4-DAP and nitrendipine (F (1,81) = 10.02, *p* = 0.0022)). Post hoc simple main effect analysis showed a significantly increased mEPP frequency in the vehicle (*peach*, ∗∗*p* = 0.0090) but not the nitrendipine (*red*, *p* = 0.3004) condition. *G*, the 1.5-μM dose of 3,4-DAP did not significantly alter mEPP amplitude in vehicle (*peach*) or nitrendipine (*red*) conditions; two-way mixed ANOVA (no significant main effect of nitrendipine; F (1,81) = 0.08568, *p* = 0.7705, or 3,4-DAP; F (1,81) = 0.2994, *p* = 0.5857, or significant interaction between 3,4-DAP and nitrendipine; F (1,81) = 2.45, *p* = 0.1214). *H* and *I*, the 1.5-μM dose of 3,4-DAP increased EPP amplitude, shown as individual pairs (*gray dotted lines*) with a superimposed average (*solid peach line*, vehicle, *H*; *solid red line*, nitrendipine, *I*); Wilcoxon signed-rank test, ∗∗∗*p* < 0.0001. 3,4-DAP, 3,4-diaminopyridine; Cav, voltage-gated calcium; EPP, endplate potential; mEPP, miniature EPPs; NMJ, neuromuscular junction.

We first evaluated the effects of 1.5-μM 3,4-DAP at the mouse NMJ (Fig. 5) because mouse NMJs are phylogenetically similar in structure and calcium channel subtype expression to human NMJs. We measured the QC (the number of vesicles released in response to an AP and calculated as the corrected and normalized mean EPP amplitude divided by corrected and normalized mean mEPP amplitude) in vehicle or nitrendipine conditions because it has been previously hypothesized that 3,4-DAP might increase neurotransmission *via* a direct effect on Cav1 channels to increase calcium flux (41, 51).

We first evaluated the effects of 1.5-μM 3,4-DAP in vehicle-treated mouse NMJs (Fig. 5). After exposure to ω-agatoxin IVA, the baseline QC averaged approximately 14 (14.2 ± 7.0; Fig. 5, *C* and *D*), whereas the mean mEPP amplitude was 0.4 ± 0.2 mV, and nerve stimulation produced a mean baseline EPP of 5.2 ± 1.8 mV (Fig. 5, *G* and *H*). Bath application of a therapeutic concentration of 3,4-DAP (1.5 μM) to vehicle-treated NMJs increased EPP amplitudes by approximately 3.2-fold (16.3 ± 5.2 mV; Fig. 5*H*), without altering mEPP amplitudes (0.4 ± 0.2 mV; Fig. 5*G*) and increased QC to approximately 44 quanta per trial (44.1 ± 15.3; a 3.1-fold increase; Fig. 5, *C* and *D*). These results are similar to 3,4-DAP effects reported previously in LEMS model mice (6, 67) and in pharmacological conditions with low probability of release (68, 69).

After ω-agatoxin IVA block in the presence of nitrendipine, the mean baseline EPP amplitude averaged 4.8 ± 2.0 mV, the mean mEPP amplitude averaged 0.4 ± 0.1 mV (Fig. 5, *G* and *I*), and the resulting QC was 11.7 ± 5.0; Fig. 5, *C* and *E*). After application of 1.5-μM 3,4-DAP, the mean EPP amplitude increased about 3.7-fold (18.5 ± 10.8 mV), without altering mEPP amplitude (0.4 ± 0.2 mV; Fig. 5, *G* and *I*), and QC increased 3.6-fold (QC = 41.6 ± 18.9; Fig. 5, *C* and *E*). The presence of nitrendipine did not significantly alter the effects of 1.5-μM 3,4-DAP on QC (Fig. 5*C*), indicating that antagonism of Cav1 channels did not alter the effects of a therapeutic concentration (1.5 μM) of 3,4-DAP at mouse NMJs. However, we did observe a significant increase in mEPP frequency in the vehicle condition, but not the nitrendipine condition (vehicle: baseline = 129.3 ± 44.9, post-3,4-DAP = 153.3 ± 49.3; nitrendipine: baseline = 153.3 ± 38.1; post-3,4-DAP = 143 ± 70.9; values are number of events per 30 s; Fig. 5*F*).

**Figure 6:**
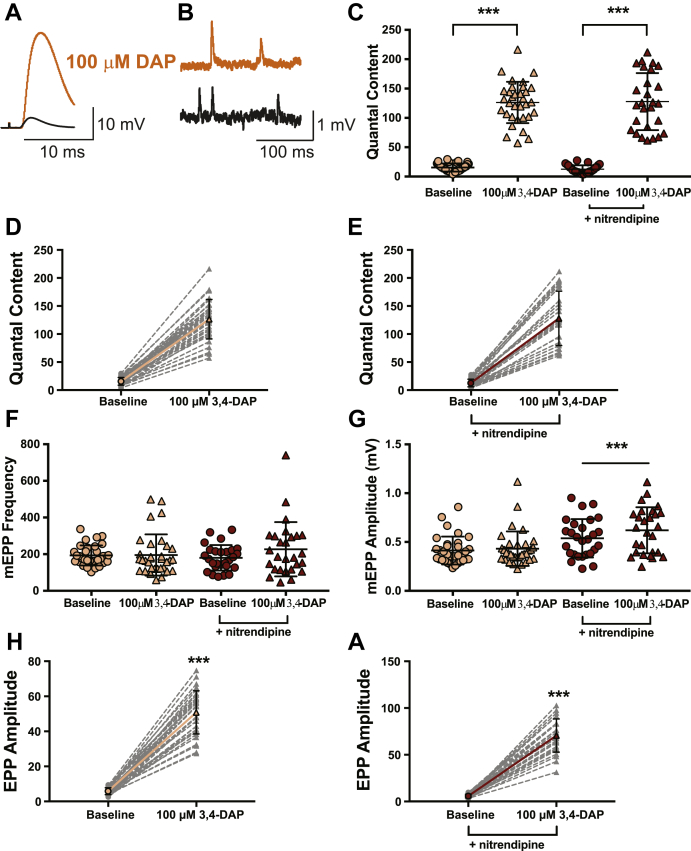
**100-μM 3,4-DAP dose dependently increases neuromuscular transmission independent of Cav1 channels in mouse neuromuscular junctions.***A* and *B*, sample traces of electrophysiological recordings of EPPs (*A*) and mEPPs (*B*) before and after 100-μM 3,4-DAP application. *C*, quantified quantal content before (*circles*) or after (*triangles*) 100-μM 3,4-DAP application to vehicle (*peach*) or nitrendipine (*red*) treated mouse NMJs. Two-way mixed ANOVA was used (there was a significant main effect of 100-μM 3,4-DAP (F (1, 58) = 548, ∗∗∗*p* < 0.0001); no significant main effect of nitrendipine (F (1,58) = 0.01559, *p* = 0.9011) or interaction between 3,4-DAP and nitrendipine (F (1,58) = 0.2182, *p* = 0.6421); vehicle, *n* = 33; nitrendipine, *n* = 27). *D* and *E*, plots of individual paired values (*gray dotted lines*) with a superimposed average (*solid peach line*, vehicle; *solid red line*, nitrendipine). *F*, the 100-μM dose of 3,4-DAP did not alter mEPP frequency in the vehicle (*peach*) or nitrendipine (*red*) condition; two-way mixed ANOVA (no main effect of nitrendipine; F (1,58) = 0.1773, *p* = 0.4209, or 3,4-DAP; F (1,58) = 2.523, *p* = 0.1176, or significant interaction between 3,4-DAP and nitrendipine; F (1,58) = 2.051, *p* = 0.1575). *G*, there was a significant interaction between the effects of 100-μM 3,4-DAP and nitrendipine on mEPP amplitude (two-way mixed ANOVA: significant main effect of nitrendipine; F (1,58) = 10.91, *p* = 0.0016) and 3,4-DAP; F (1,58) = 19.03, *p* < 0.0001) and a significant interaction between 3,4-DAP and nitrendipine; F (1,58) = 7.380, *p* = 0.0087)). Post hoc simple main effect analysis showed a significantly altered mEPP amplitude in the nitrendipine (*red*, ∗∗∗*p* < 0.0001) but not vehicle condition (*peach*, *p* = 0.4497). The 100-μM dose of 3,4-DAP increased EPP amplitude, shown as individual pairs (*gray dotted lines*) with a superimposed average (*solid peach line*, vehicle, *H*; *solid red line*, nitrendipine, *I*); Wilcoxon signed-rank test, ∗∗∗*p* < 0.0001. 3,4-DAP, 3,4-diaminopyridine; Cav, voltage-gated calcium; EPP, endplate potential; mEPP, miniature EPPs; NMJ, neuromuscular junction.

Next, we evaluated the effects of a supratherapeutic concentration of 3,4-DAP (100 μM) in vehicle or nitrendipine-treated, 3,4-DAP–naïve mouse NMJ preparations (Fig. 6). In NMJs treated with the vehicle, the mean mEPP amplitude was 0.4 ± 0.1 mV, and motor nerve stimulation produced a mean baseline EPP amplitude of 5.8 ± 1.9 mV after exposure to ω-agatoxin IVA (Fig. 6, *G* and *H*). Quantal release under these conditions was approximately 16 (15.5 ± 6.9; Fig. 6, *C* and *D*). After exposure to 100-μM 3,4-DAP, the mean EPP amplitude was significantly increased by approximately 8.8-fold (50.8 ± 12.3 mV), without a significant change in the mean mEPP amplitude (0.4 ± 0.2 mV; Fig. 6, *G* and *H*). This resulted in an increase in QC from 15.5 quanta per trial to approximately 126 quanta per trial (126.3 ± 35.2), which is an 8.1-fold increase in quantal release (Fig. 6, *C* and *D*).

We next assessed whether nitrendipine could alter the effects of a supratherapeutic concentration of 3,4-DAP (100 μM). After ω-agatoxin IVA treatment in the presence of nitrendipine, EPP amplitude averaged 5.7 ± 2.0 mV and mEPP amplitude averaged 0.5 ± 0.2 mV (Fig. 6, *G* and *I*) and this resulted in a QC of 12.5 ± 6.8 (Fig. 6, *C* and *E*). Bath application of 100-μM 3,4-DAP increased EPP amplitude by about 12.3-fold (70.5 ± 17.8 mV), and mEPP amplitude averaged 0.6 ± 0.2 mV (Fig. 6, *G* and *I*). QC significantly increased to 127.8 ± 48.6 after application of 100-μM 3,4-DAP, or a 10.2-fold increase (Fig. 6, *C* and *E*). We did not observe a significant effect of nitrendipine on QC (Fig. 6*C*) or mEPP frequency (vehicle: baseline = 193.2 ± 54.8, post-3,4-DAP = 195.6 ± 112.4; nitrendipine: baseline = 179.9 ± 69.4; post-3,4-DAP = 226.7 ± 148.1; values are the number of events per 30 s; Fig. 6*F*). However, we did observe a significant effect of 3,4-DAP on mEPP amplitude in the nitrendipine condition (Fig. 6*G*).

**Figure 7:**
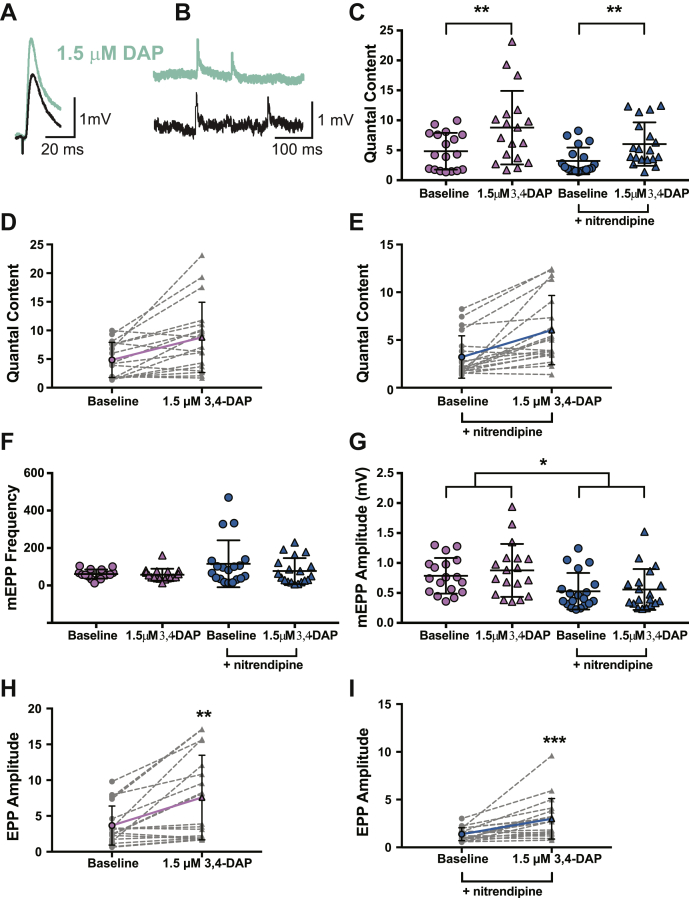
**1.5-μM 3,4-DAP dose dependently increases neuromuscular transmission independent of Cav1 channels in frog neuromuscular junctions.***A* and *B*, sample traces of electrophysiological recordings of EPPs (*A*) and mEPPs (*B*) before and after 1.5-μM 3,4-DAP application. *C*, quantified quantal content before (*circles*) or after (*triangles*) 1.5-μM 3,4-DAP application to vehicle (*pink*) or nitrendipine (*blue*) treated frog NMJs. Two-way mixed ANOVA was used (there was a significant main effect of 1.5-μM 3,4-DAP (F (1, 35) = 28.62, ∗∗∗*p* < 0.0001); no significant main effect of nitrendipine (F (1,35) = 3.517, *p* = 0.0691) or a significant interaction between 3,4-DAP and nitrendipine (F (1,35) = 0.785, *p* = 0.3817); vehicle, *n* = 18; nitrendipine *n* = 19). *D* and *E*, plots of individual paired values (*gray dotted lines*) with a superimposed average (*solid pink line*, vehicle; *solid blue line*, nitrendipine). *F*, the 1.5-μM dose of 3,4-DAP did not alter mEPP frequency in the vehicle (*pink*) or nitrendipine (blue) condition; two-way mixed ANOVA (no main effect of nitrendipine; F (1,35) = 2.856, *p* = 0.0999) or 3,4-DAP; F (1,35) = 3.418, *p* = 0.0729 or a significant interaction between 3,4-DAP and nitrendipine; F (1,35) = 2.352, *p* = 0.1341). *G*, mEPP amplitude was significantly different between the vehicle (*pink*) and nitrendipine (*blue*) conditions but was not affected by 3,4-DAP; two-way mixed ANOVA (significant main effect of nitrendipine (F (1,35) = 7.019, *p* = 0.0121), and there was no significant main effect of 3,4-DAP (F (1,35) = 2.604, *p* = 0.1156) or significant interaction between 3,4-DAP and nitrendipine (F (1,35) = 0.6628, *p* = 0.4211)). *H* and *I*, the 1.5-μM dose of 3,4-DAP increased EPP amplitude, shown as individual pairs (*gray dotted lines*) with a superimposed average (*solid pink line*, vehicle, *H*; paired t test, ∗∗ *p* = 0.0012; *solid blue line*, nitrendipine, *I*; Wilcoxon signed-rank test, ∗∗∗*p* < 0.0001). 3,4-DAP, 3,4-diaminopyridine; Cav, voltage-gated calcium; EPP, endplate potential; mEPP, miniature EPPs; NMJ, neuromuscular junction.

We also tested the effect of 3,4-DAP at frog NMJs (a traditional model of neuromuscular function) to evaluate whether 3,4-DAP mechanisms of action are phylogenetically conserved and determine if nitrendipine alters 3,4-DAP effects. We first assessed the effects of 1.5-μM 3,4-DAP on vehicle-treated frog NMJs (Fig. 7). After exposure to ω-conotoxin GVIA, motor nerve stimulation evoked a mean baseline EPP amplitude of 3.7 ± 2.7 mV, and spontaneous release resulted in a mean mEPP amplitude of 0.8 ± 0.3 mV in vehicle-treated NMJs (Fig. 7, *G* and *H*), producing a QC of about 5 (QC = 4.8 ± 3.1; Fig. 7, *C* and *D*). After application of 1.5-μM 3,4-DAP, the mean EPP amplitude increased approximately 2-fold (to 7.6 ± 5.9 mV), without significantly altering mEPP amplitude (0.9 ± 0.4 mV; Fig. 7, *G* and *H*). Therefore, after exposure to 1.5-μM 3,4-DAP, QC increased to 8.8 ± 6.1 (a 1.8-fold increase; Fig. 7, *C* and *D*).

We next assessed 1.5-μM 3,4-DAP on frog NMJs in the presence of nitrendipine. After exposure to ω-conotoxin GVIA, the average baseline EPP amplitude was 1.3 ± 0.7 mV, the average mean mEPP amplitude was 0.5 ± 0.3 mV (Fig. 7, *G* and *I*), and QC was about 3 (QC = 3.2 ± 2.2; Fig. 7, *C* and *E*). After bath application of 1.5-μM 3,4-DAP, the mean EPP amplitude increased by about 2.3-fold to 3.0 ± 2.1 mV, with an average mEPP amplitude of 0.7 ± 0.3 mV (Fig. 7, *G* and *I*), which resulted in a QC of 6.0 ± 0.8 (a 1.9-fold increase; Figure C, E). We did not observe a significant effect of nitrendipine on QC (Fig. 7*C*), although we did observe a significant difference in mEPP amplitudes in vehicle-treated *versus* nitrendipine-treated NMJs (Fig. 7, *F* and *G*). Neither 3,4-DAP nor nitrendipine altered mEPP frequency (vehicle: baseline = 61 ± 24.2, post-3,4-DAP = 57.4 ± 31.9; nitrendipine: baseline = 115.6 ± 125.3; post-3,4-DAP = 76.9 ± 69.3; values are the number of events per 30 s).

**Figure 8:**
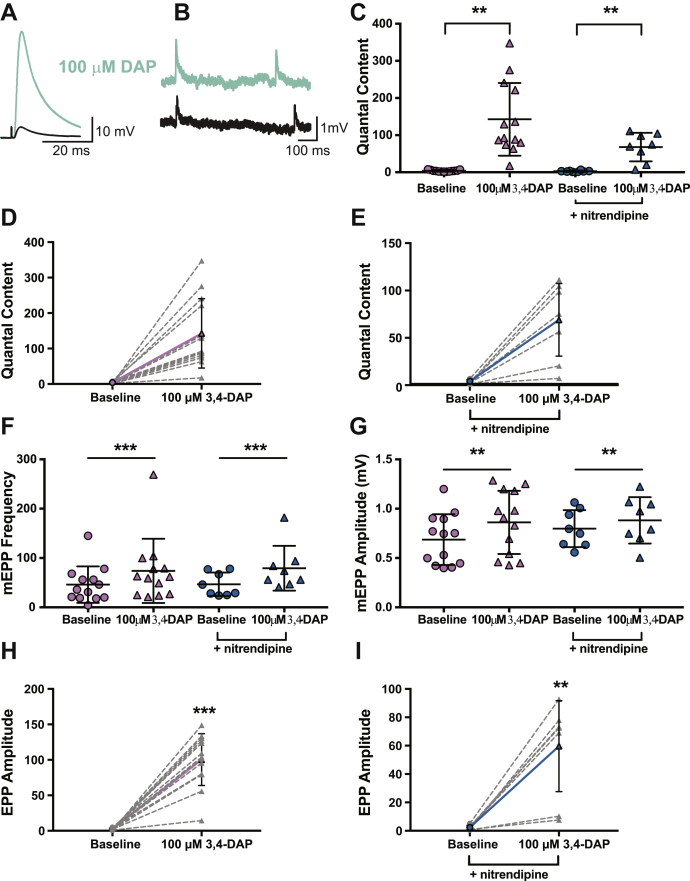
**100-μM 3,4-DAP dose dependently increases neuromuscular transmission independent of Cav1 channels in frog neuromuscular junctions.***A* and *B*, sample traces of electrophysiological recordings of EPPs (*A*) and mEPPs (*B*) before and after 100-μM 3,4-DAP application. *C*, quantified quantal content before (*circles*) or after (*triangles*) 100-μM 3,4-DAP application to vehicle (*pink*) or nitrendipine (*blue*) treated frog NMJs. Two-way mixed ANOVA was used (there was a significant main effect of 100-μM 3,4-DAP (F (1, 19) = 31.66, ∗∗∗*p* < 0.0001; no significant main effect of nitrendipine (F (1,19) = 4.226, *p* = 0.0538) nor a significant interaction between 3,4-DAP x nitrendipine (F (1,19) = 4.162, *p* = 0.0555): vehicle, *n* = 13; nitrendipine *n* = 8). *D* and *E*, plots of individual paired values (*gray dotted lines*) with a superimposed average (*solid pink line*, vehicle; *solid blue line*, nitrendipine). *F*, the 100-μM dose of 3,4-DAP increased mEPP frequency in both the vehicle (*pink*) and nitrendipine (*blue*) conditions; two-way mixed ANOVA (significant main effect of 3,4-DAP; F (1,19) = 9.541, *p* = 0.006; no main effect of nitrendipine; F (1,19) = 0.0264, *p* = 0.8727) or a significant interaction between 3,4-DAP and nitrendipine; F (1,19) = 0.0568, *p* = 0.8143). *G*, the 100-μM dose of 3,4-DAP significantly altered mEPP amplitude in both the vehicle (*pink*) and nitrendipine (*blue*) conditions; two-way mixed ANOVA (significant main effect of 3,4-DAP; F (1,19) = 11.88, *p* = 0.0027, but not nitrendipine; F (1,19) = 0.3468, *p* = 0.5628, and no significant interaction between 3,4-DAP and nitrendipine; F (1,19) = 1.436, *p* = 0.2454). *H* and *I*, the 100-μM dose of 3,4-DAP increased EPP amplitude, shown as individual pairs (*gray dotted lines*) with a superimposed average (*solid pink line*, vehicle, *H*; *solid blue line*, nitrendipine, *I*); paired t test, ∗∗*p* = 0.0012, ∗∗∗*p* < 0.0001. 3,4-DAP, 3,4-diaminopyridine; Cav, voltage-gated calcium; EPP, endplate potential; mEPP, miniature EPPs; NMJ, neuromuscular junction.

Next, we assessed the effects of 100-μM 3,4-DAP on frog NMJs in the presence of vehicle (Fig. 8). Before 3,4-DAP exposure, motor nerve stimulation produced an average EPP amplitude of 2.4 ± 1.2 mV, and spontaneous release resulted in an average mean mEPP amplitude of 0.7 ± 0.3 mV (Fig. 8, *G* and *H*), resulting in a QC of about 4 (QC = 3.8 ± 2.3; Fig. 8, *C* and *D*). After 100-μM 3,4-DAP treatment, the EPP amplitude increased to an average of 100.2 ± 36.5 mV (Fig. 8 *H*), and the mEPP amplitude was 0.9 ± 0.3 mV (Fig. 8 *G*). This resulted in a significant increase in QC to 142.6 ± 97.9 (a 37.5-fold increase; Fig. 8, *C* and *D*).

We subsequently assessed whether nitrendipine could modulate 100-μM 3,4-DAP effects on neurotransmission at frog NMJs. For these experiments, the average baseline EPP amplitude in the presence of nitrendipine was 2.3 ± 1.5 mV, with an average mEPP amplitude of 0.8 ± 0.2 mV (Fig. 8, *G* and *I*), resulting in a QC of about 3 (QC = 2.9 ± 1.8; Fig. 8, *C* and *E*). After bath application of 100-μM 3,4-DAP in the presence of nitrendipine, EPP amplitude increased to 59.6 ± 32.1 mV, the average mEPP amplitude was 0.9 ± 0.2 mV (Fig. 8*G*), resulting in a QC of about 68 (QC = 67.8 ± 38.3, a 23.4-fold increase; Fig. 8, *C* and *E*). We did not observe an effect of nitrendipine on 3,4-DAP–induced changes in QC. Therefore, there was no significant difference between the effect of 100-μM 3,4-DAP in the absence or presence of nitrendipine (Fig. 8*C*). However, we did find that 100-μM 3,4-DAP increased mEPP frequency regardless of vehicle or nitrendipine condition (vehicle: baseline = 46 ± 37.0, post-3,4-DAP = 73.9 ± 65.0; nitrendipine: baseline = 46.8 ± 23.8; post-3,4-DAP = 79.3 ± 45.4; values are the number of events per 30 s; Fig. 8, *F* and *G*). Therefore, we found that 100-μM 3,4-DAP increased mEPP frequency and amplitude regardless of vehicle or nitrendipine condition.

These data, taken together with the effect of low micromolar concentrations of 3,4-DAP on Kv3 channels (Fig. 1) and AP waveforms (Fig. 2), lead us to conclude that low micromolar concentrations of 3,4-DAP enhance the magnitude of AP-evoked transmitter release at mammalian NMJs by partial antagonist activity on Kv3 channels in the motor nerve terminal, which broaden the presynaptic AP without directly affecting Cav1 channels. Furthermore, Cav1 channels do not significantly contribute to the effects on AP waveforms or QC at the NMJ of a higher concentration (100 μM) of 3,4-DAP.

## Discussion

3,4-DAP is the FDA-approved first-line treatment for patients with Lambert–Eaton Myasthenic Syndrome (LEMS; (7, 8, 9, 10)). Although 3,4-DAP is canonically thought to mediate its effects by partially blocking presynaptic Kv channels, a previous report that millimolar concentrations of 3,4-DAP could have off-target effects on Cav1 channels led to the question of whether 3,4-DAP mechanisms of action at therapeutic concentrations (estimated to be in the low micromolar range) involve Cav1 channels. For this reason, we sought to characterize the effects of 3,4-DAP on Kv3 potassium current, the presynaptic AP waveform, and transmitter release to determine if effects on AP waveforms and transmitter release were altered after using nitrendipine to block Cav1 (L-type) calcium channels. We found that the effects of 3,4-DAP on AP-evoked transmitter release at low micromolar concentrations could be explained by a partial block of Kv3 channels that results in the broadening of the presynaptic AP independent of any contribution from Cav1 calcium channels.

We did observe some small but significant effects of 3,4-DAP on mEPP frequency and amplitude, which may be due to as yet undetermined presynaptic and/or postsynaptic effects. It is possible that high doses of 3,4-DAP are not selective for presynaptic Kv3 channels and affect postsynaptic Kv channels to indirectly modulate acetylcholine receptor sensitivity. In addition, the large amount of calcium influx into motor nerve terminals induced by 100-μM 3,4-DAP may alter normal homeostatic mechanisms of calcium buffering and handling, resulting in increased spontaneous vesicle release. Although it is also unclear how nitrendipine might alter the effects of 3,4-DAP on mEPPs in mouse and frog NMJs, it is evident that the change in the magnitude of neurotransmission (QC) induced by 3,4-DAP is unaffected by Cav1 channels.

Previously, 4-aminopyridine has been reported to have IC_50_ values for blocking Kv3 channels of 30 μM to 2.5 mM (depending in the Kv subtype; (37, 38, 39, 40, 41, 42, 43, 44, 45, 46)), and although 3,4-DAP effects on the squid giant axon potassium channel (SqKv1A; (70)) have been reported in the low micromolar range (50), we are not aware of any prior study characterizing the concentration-dependent effects of 3,4-DAP on the Kv3 subtype of potassium channels. Although we found that 3,4-DAP could strongly inhibit Kv3 channels in the high micromolar to millimolar range with IC_50_ values of 150 to 250 μM (which we defined as low affinity binding), we remarkably discovered distinct high-affinity effects of 3,4-DAP on Kv3 channels. We have shown that 3,4-DAP has similar effects on Kv3.3 and Kv3.4 and acts as a partial antagonist, binding in the low micromolar range (IC_50_ = 2.5–10 μM). In particular, Kv3.3 and Kv3.4 are both blocked by about 10% after exposure to the therapeutically relevant concentration of 1.5-μM 3,4-DAP. Thus, we predict that even a 10% decrease in Kv3.3 or Kv3.4 current would have a significant effect on the AP duration in motor nerve terminals.

Kv3 currents have been shown to mediate the dominant outward current during brief AP depolarizations within many nerve terminals (71, 72, 73, 74) and have also been shown to have rapid activation and inactivation characteristics that enable nerve terminals to fire APs with short duration and at high frequency (75, 76, 77). These data are consistent with the AP waveforms that were recently optically recorded from frog presynaptic nerve terminals (64). Here, we report concentration-dependent broadening of the presynaptic AP at the frog and mouse NMJ after exposure to 3,4-DAP. Even relatively small changes in presynaptic AP duration (15–20%) have been predicted to have significant effects on calcium ion entry and transmitter release (64). Thus, the roughly 20% broadening reported here at the mouse NMJ would be predicted to underlie the approximate 3-fold increase in transmitter release we observed (64). Interestingly, we found that 1.5-μM 3,4-DAP broadened the presynaptic AP waveform in the frog NMJ to a greater extent than at the mouse NMJ. A potential species difference in presynaptic ion channel subtype expression and/or density may underlie these results and requires further investigation.

At neuromuscular synapses, the very brief presynaptic AP waveform only activates a small percentage of the Cav2 channels positioned within transmitter release sites (78, 79). This is thought to ensure that each of the hundreds of transmitter release sites within a single NMJ releases transmitter with low probability, conserving resources for repeated activation during normal activity (80). A brief AP activating only a small subset of available presynaptic Cav2 channels leads to neuromuscular weakness after many of these calcium channels are attacked and removed by autoantibodies in the disease LEMS. Mechanistically, 3,4-DAP is an effective symptomatic treatment for LEMS because the partial block of presynaptic Kv3 channels broadens the presynaptic AP, which increases the percentage of presynaptic Cav channels that open, and thus increases calcium entry and calcium-triggered transmitter release, leading to an improvement in neuromuscular strength in patients with LEMS.

## Experimental procedures

### Ethics statement

The experimental procedures in this study were conducted in compliance with the US National Institutes of Health laboratory animal care guidelines and approved by the Institutional Animal Care and Use Committee of the University of Pittsburgh. All efforts were made to minimize the suffering of animals.

### Whole-cell perforated patch-clamp electrophysiology

Recordings were performed as described previously (81) using HEK293T cells transfected with Kv3.3 (Kv 3.3 α subunit (Dr Leonard Kaczmarek) and GFP at a DNA ratio of 1:1), Kv3.4 (Kv 3.4 α subunit (Dr Manuel Covarrubias) and GFP at a DNA ratio of 1:1), Cav2.1 (Cav 2.1 α1 subunits, Addgene #26573; Cav β3, Addgene #26574; Cav α2δ1, Addgene #26575, and GFP at a DNA ratio of 1:1:1:1), or Cav1.2 (Cav 1.2 α1 subunits, Addgene #26572; Cav β3, Addgene #26574; Cav α2δ1, Addgene #26575, and GFP at a DNA ratio of 1:1:1:1). All recordings were performed at room temperature (RT) (20–22 °C).

For recording potassium currents, the pipette solution contained 70-mM CH_3_KO_3_S, 60-mM KCl, 10-mM Hepes, and 1-mM MgCl_2_ at pH 7.4, and the bath saline contained 130-mM NaCl, 10-mM Hepes, 10-mM glucose, 3-mM CaCl_2_, and 1-mM MgCl_2_ at pH 7.4. For recording calcium currents, the pipette solution contained 70-mM CH_3_O_3_SCs, 60-mM CsCl, 10-mM Hepes, and 1-mM MgCl_2_ at pH 7.4, and the bath saline contained 130-mM choline chloride, 10-mM Hepes, 10-mM TEA-Cl, 5-mM BaCl_2_, and 1-mM MgCl_2_ at pH 7.4. Patch pipettes were fabricated from borosilicate glass and pulled to a resistance of ∼1 to 4 MΩ. Before each experiment, 3-mg amphotericin-B was dissolved in 50-μM dimethyl sulfoxide. Each hour, 10 μl of this amphotericin-B stock was mixed with 500 μl of the pipette solution and vortexed. Pipettes were filled in a two-step process. The tip of the pipette was dipped into a droplet of filtered pipette solution for 1 s, and then the remainder of the pipette was back-filled with the amphotericin-B/pipette solution mixture using a syringe and a 34 G quartz needle (MicroFil MF34G, World Precision Instruments). This filled pipette was then used to make a GΩ seal with a fluorescent cell and 5 to 10 min was provided for amphotericin-B–mediated perforated patch access. Access resistances ranged from 5 to 15 MΩ and were compensated by 85%. Voltage clamp of cells under study was controlled by an Axopatch 200B amplifier driven by Clampex 10 software (Molecular Devices). Data were filtered at 5 kHz, digitized at 10 or 50 kHz, and analyzed using Clampfit 10 software (Molecular Devices). Capacitive transients and passive membrane responses to the voltage steps were subtracted, and the liquid junction potential was corrected before each recording. Current through calcium or potassium channels was activated by depolarizing steps from a holding potential of −100 mV to +20 or +40 mV. In all cases, currents were activated both before and after exposure to 3,4-DAP (dissolved in extracellular saline) in each cell, and current amplitudes were compared. For analysis before plotting, each current amplitude was normalized to its peak current amplitude before 3,4-DAP application to derive the normalized block at each concentration. The ratio described above was subtracted from 1 and the resulting value then was multiplied by 100 to obtain the percent inhibition.

### Tissue preparation

Adult male and female frogs (*Rana pipiens*) were anaesthetized *via* immersion in 0.6% tricaine methane sulphonate, decapitated, and double pithed. The cutaneous pectoris neuromuscular preparation was dissected and bathed in normal frog Ringer saline (in mΜ: 116 NaCl, 10-mM N,N-bis(2-hydroxyethyl)-2-aminoethanesulfonic acid (BES) buffer, 2-mM KCl, 5-mM glucose, 1-mM MgCl_2_, 1.8-mM CaCl_2_, pH 7.3). Adult male and female Swiss Webster mice (3–6 months of age; Charles River Laboratories) were sacrificed using CO_2_ inhalation, followed by thoracotomy. The epitrochleoanconeous neuromuscular preparation was bilaterally dissected and bathed in normal mammalian Ringer saline (in mΜ: 150 NaCl, 10-mM BES buffer, 5-mM KCl, 11-mM glucose, 1-mM MgCl_2_, 2-mM CaCl_2_, pH 7.4).

### Intracellular microelectrode electrophysiology

The muscle nerve was stimulated using a suction electrode, and muscle contraction was blocked after 1-h incubation in a bath containing 50 μM of the irreversible muscle myosin inhibitor 3-(N-butylethanimidoyl)-4-hydroxy-2H-chromen-2-one (82). After 3-(N-butylethanimidoyl)-4-hydroxy-2H-chromen-2-one washout using normal saline, microelectrode recordings were made in the presence of 1-μM nitrendipine (Sigma) or the vehicle (0.01% dimethyl sulfoxide) plus a selective muscle voltage-gated sodium channel blocker (1-μM μ-conotoxin PIIIA for the frog NMJ or 5-μM μ-conotoxin GIIIB for the mouse NMJ; Alomone Labs Ltd). In addition, to reduce the magnitude of transmitter released, 250- to 900-nM ω-conotoxin GVIA (to block N-type channels at the frog NMJ) or 50- to 100-nM ω-agatoxin IVA (to block P/Q-type channels at the mouse) was included in the recording bath. The range of concentrations listed was used iteratively with each preparation to reduce control EPPs to below 10 mV. Intracellular recordings of muscle cell membrane potentials were obtained using borosilicate glass microelectrodes pulled to a resistance of ∼40 to 60 MΩ and filled with 3-M potassium chloride. For each muscle fiber recording made adjacent to visualized NMJs, spontaneous miniature synaptic events (mEPPs) were collected for 1 to 2 min, followed by 10 to 30 EPPs elicited by low-frequency (0.2 Hz) nerve stimulation. Subsequently, neuromuscular preparations were incubated in freshly made 1.5-μM or 100-μM 3,4-DAP for 30 to 60 min. After 3,4-DAP incubation, paired recordings were made from the same NMJs that had been studied in control saline (resulting in paired data sets). Data were collected using an Axoclamp 900A and digitized at 10 kHz for analysis using pClamp 10 software (Molecular Devices). Spontaneous and evoked EPPs were normalized to −70 mV and corrected for nonlinear summation (65). We measured the magnitude of transmitter release by determining the QC using the direct method of dividing the peak of the averaged and normalized EPP trace by the peak of the averaged and normalized mEPP trace.

### Voltage imaging

Voltage imaging was performed as described previously (64). To load nerve terminals with the dye for the voltage-imaging procedure, a mixture of 5 ml of normal frog Ringer saline (for frog preparations) or normal mammalian Ringer saline (for mouse preparations) with a BeRST 1 voltage-sensitive dye (63) concentration of 0.5 μM and 10 μg/ml of Alexa Fluor 488–conjugated alpha-bungarotoxin (BTX; to counterstain postsynaptic receptors at the NMJ and block muscle contractions) was freshly made before each experiment. Then, the neuromuscular preparation was bathed in this dye mixture for 90 min, rinsed, and mounted on the stage of an Olympus BX61 microscope with a 60x water immersion objective. The nerve was then drawn into a suction electrode for suprathreshold stimulation. If the BTX conjugated to Alexa Fluor 488 did not completely block muscle contractions, 10-μM curare was added to the imaging saline to completely block any remaining nerve-evoked muscle contractions.

The postsynaptic BTX stain was used to identify nerve terminals and bring them into focus for voltage imaging. After locating a well-stained nerve terminal, an imaging region of interest (ROI) that contained a large portion of the nerve terminal branch (usually an ROI of approximately 80 × 30 μm for frog or 60 × 40 μm for mouse) was selected. All voltage imaging recordings were performed at RT (20–22 °C).

After a nerve terminal was selected for imaging, the presynaptic axon was stimulated at 0.2 Hz. During each stimulation, there was a brief 100-μs image collection window where the preparation was illuminated by a 640-nm laser (89 North laser diode illuminator) and the BeRST 1 dye fluorescence of the nerve terminal was recorded by an EMCCD camera (Pro-EM 512, Princeton Instruments). A custom routine on a Teensy 3.5 USB development board (PJRC) created a delay between the stimulation of the nerve and the triggering of the camera and laser. This delay in the 100-μs collection window was increased by 20 μs after each stimulation. After 100 sequential delays of 20 μs, a full time-course of 2 ms in which the entire AP waveform could be sampled was obtained (for 100-μM 3,4-DAP recordings, 300 moving bins for a total time course of 6 ms were used). For each frog nerve terminal recording, this process was repeated 5 to 15 times. For mouse nerve terminal recordings, this process was repeated 10 to 50 times (the BeRST 1 dye signal at the mouse terminals was weaker than at the frog terminals and thus required more recordings to obtain a high-quality averaged AP waveform).

Custom-written scripts in ImageJ and MATLAB (Mathworks) were used to analyze images. An “align slices in stack” ImageJ plugin (https://sites.google.com/site/qingzongtseng/template-matching-ij-plugin; see (83, 84)) was used to correct the image stack for x-y drift. Then, an unbiased ROI selection (a subsection of the full imaging ROI) containing the nerve terminal was created by applying an Otsu local imaging threshold (85) to the average fluorescence z-projection of the BeRST 1 image stack. The average fluorescence inside this ROI was used as the nerve signal (for frog recordings, 20 μm near the end of the nerve terminal and last node of Ranvier were excluded to restrict recordings to the middle electrical region of the terminal; see (64)). The average fluorescence from the region outside of the Otsu-selected ROI was used as the background signal. Both the nerve and background signals were then low-pass filtered offline (f_pass_= 4 kHz). The following analysis was then performed separately for both the filtered and unfiltered signals: the background signal was divided from the nerve signal to generate a corrected fluorescence signal. We then fit a cubic B-spline through the unstimulated points in the fluorescence time course (the first and last 15 points of the 100 total points in each time series), and divided this cubic spline from each point in the fluorescence signal. This resulted in a ΔF/F fluorescence signal that did not fluctuate as a result of drift of the nerve muscle preparation or dye bleaching.

Two AP waveforms were then created by separately averaging the APs from the filtered and unfiltered ΔF/F fluorescence signal. The R^2^ value between the filtered and unfiltered AP waveforms was then calculated. Because these are not linear models, the R^2^ is not an exact measure of fit between the filtered and unfiltered AP waveforms but rather is a metric of fit that is heavily weighted by the strength (in terms of the amplitude of the ΔF/F fluorescence signal) of the recorded signal. This weight is important because normalizing slight bumps on an almost flat signal could appear as an AP. Thus, the R^2^ value provides a heuristic metric to estimate the quality of our recorded AP waveforms and is not used for any statistical purposes.

Image artifacts in the background (*e.g.*, a BeRST 1 dye stained free-floating piece of connective tissue) occasionally resulted in the background not properly dividing the nerve signal, resulting in a noisier signal (and worse R^2^ value). If the R^2^ was less than 0.95 for frog recordings (or 0.90 for the 300 bin recordings for the 100-μM 3,4-DAP recordings), or 0.85 for the mouse recordings, the fluorescence of an approximately 15 × 30-μm section of the background near the nerve terminal was used as the background fluorescence rather than the complete background region. If this smaller background subsection also resulted in an R^2^ value lower than the values listed above, the recording was not included in the data analysis. If the recording was of high enough quality to produce a sufficient R^2^ value, the average filtered AP was normalized to the first 15 points (the baseline of the trace) and fit with cubic spline interpolation at an oversampled time resolution of 2 μs. Finally, the FWHM of the normalized spline of the AP waveform was calculated.

### Statistical analysis

Data were statistically analyzed using Prism v.7 or v.8 (Graphpad). Electrophysiology data were determined to be outliers if the data exceeded 1.5 times the interquartile range. The distribution of the data was assessed for normality using the Shapiro–Wilk test. Statistical comparisons were performed using a two-way repeated-measures mixed ANOVA with a between-subject factor of bath (nitrendipine or control vehicle bath) and a within-subject factor of 3,4-DAP (baseline or after 3,4-DAP bath application), or with a paired t test or Wilcoxon matched-pairs signed-rank test. If there was a significant interaction found, post hoc simple effect tests were performed. Results were considered statistically significant when the *p*-value was <0.05. The results represent the mean ± standard deviation of at least three independent experiments.

## Data availability

Data described in the manuscript are all data contained within the manuscript and can be shared upon request to Stephen D. Meriney, meriney@pitt.edu.

## Conflict of interest

The authors declare that they have no conflicts of interest with the contents of this article.
